# Cyclic negative pressure promotes chondrocyte growth: Association of IGF-II with EGR-1

**DOI:** 10.17305/bb.2024.10487

**Published:** 2024-12-01

**Authors:** Xiaoyu Li, Lixia Huang, Bingxue Liu, Zehua Zhang, Guoyong Zhou, Zirui Guo, Jiuhong Song, Xiang Wang

**Affiliations:** 1 School of Medicine, Jianghan University, Wuhan, China; 2 Tianyuan Translational Medicine Research and Development Team, School of Medicine, Jianghan University, Wuhan, China; 3 Institute of Translational Medicine, Wuhan College of Arts and Science, Wuhan, China; 4 Department of Materials, Swiss Federal Institute of Technology, Zurich, Switzerland; 5 Wuhan FL Medical Science and Technology Ltd., Wuhan, China

**Keywords:** Osteoarthritis, chondrocytes, biomechanics, cell proliferation, early growth response 1 (EGR-1), insulin-like growth factor 2 (IGF-II)

## Abstract

Knee osteoarthritis (KOA) is one of the most common degenerative joint diseases in the elderly worldwide. The primary lesion in patients with KOA is the degeneration of articular cartilage. This study aimed to observe the biological effects of cyclic negative pressure on C28/I2 chondrocytes and to elucidate the underlying molecular mechanisms. We designed a bidirectional intelligent micro-pressure control device for cyclic negative pressure intervention on C28/I2 chondrocytes. Chondrocyte vitality and proliferation were assessed using Cell Counting Kit-8 (CCK-8) and 5-ethynyl-2’-deoxyuridine (EdU) assays. The extracellular matrix was analyzed using real-time fluorescence quantitative polymerase chain reaction (PCR) and western blot, while the molecular mechanism of the chondrocyte response to cyclic negative pressure was explored through mRNA sequencing. Experimental data demonstrated that cyclic negative pressure promoted chondrocyte proliferation and upregulated the expression of chondrocyte-specific protein, namely, the collagen type II alpha 1 chain (COL2A1) protein, and the transcription factor SRY-box transcription factor 9 (SOX9). Additionally, RNA sequencing analysis revealed that the gene levels of insulin-like growth factor 2 (*IGF-II*) and early growth response 1 (*EGR-1*) were significantly elevated in the cyclic negative pressure group (NP Group). This study demonstrates that cyclic negative pressure stimulates the proliferation of C28/I2 chondrocytes by promoting the expression of *EGR-1* and *IGF-II*. This new discovery may provide novel insights into cartilage health and KOA prevention.

## Introduction

Knee osteoarthritis (KOA) is a total knee disorder that typically involves the articular cartilage, meniscus, infrapatellar fat pad, synovium, and subchondral bone [1, 2]. During disease progression, the primary tissue affected is usually the articular cartilage, which mainly consists of chondrocytes [3]. In the early stages of KOA, the articular cartilage begins to deteriorate, gradually progressing to deeper layers and surrounding tissues, eventually resulting in severe damage to the joint structure and function [4]. This process is chronic, progressive, and irreversible [5]. Therefore, exploring the mechanisms of articular cartilage degradation is essential for the prevention and early treatment of KOA to slow down the progression of the disease and preserve joint function.

Significant attention is focused on the fact that chondrocytes are in a unique stress environment within the joint. Inside the joint, they are subjected to mechanical loads, primarily from hydrostatic pressure, which is generated by the water pressure within the cartilage space. They also experience tensile forces from neighboring tissues, as well as static and dynamic stresses from changes in stance or posture [6–8]. Some in vitro studies showed that different pressures can cause opposite effects in chondrocytes, including increased apoptosis and decreased apoptosis [9–12]. These studies used positive pressures higher than atmospheric pressure as the intervention condition. However, the pressure range in the normal human knee joint in a fully relaxed state is about −10 to 0 mmHg, which is lower than the atmospheric pressure [13]. This means that physiologically, the human knee cartilage is in a negative pressure environment most of the time. No research has been conducted on the effect of a negative pressure environment on chondrocytes so far.

**Figure 1.**
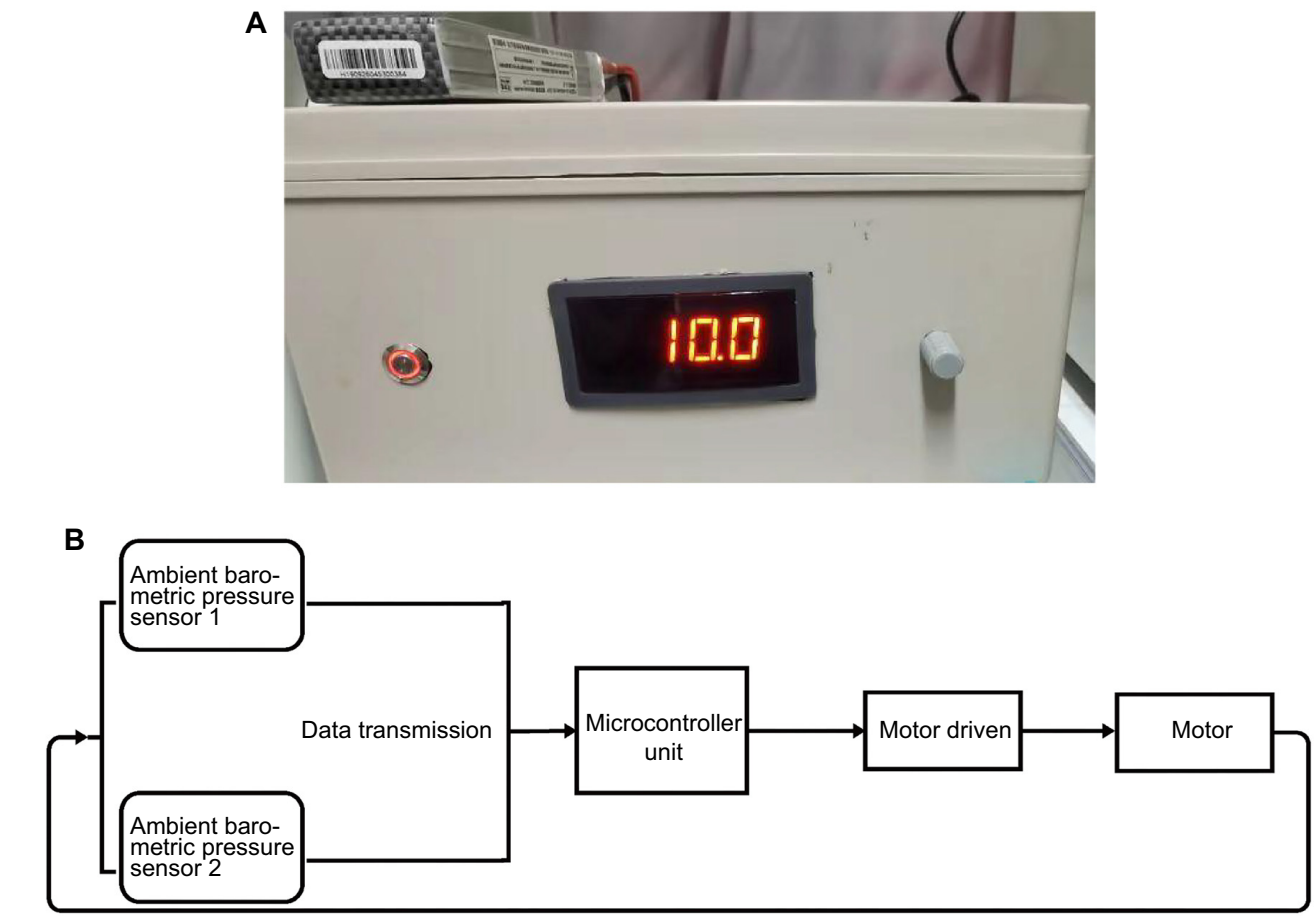
**Cyclic negative pressure equipment and schematic diagram.** (A) Exterior view of the cyclic negative pressure equipment; (B) Cyclic negative pressure equipment operating schematic.

A clinical trial conducted by our team in 2021 showed that the resting state knee joint cavity pressure in the supine position in healthy volunteers was −11.32 ± 0.21 cm H_2_O, while KOA volunteers recorded a much higher pressure of −3.52 ± 0.34 cm H_2_O [14]. It was also observed in the same trial that this negative pressure varied cyclically during simulated walking in hip flexion and posterior extension maneuvers. Based on the above studies, we hypothesized that the deterioration of chondrocytes and, thus, the occurrence of KOA may be related to an altered negative pressure environment in the knee joint cavity and that restoring the negative pressure environment to that of a healthy knee joint cavity may promote chondrocyte growth.

Cell changes in the early environment include the activation of early genes. When cells are stimulated, early growth response factors respond rapidly. Early growth response 1 (*EGR-1*) is a member of the early growth-responsive gene family. *EGR-1* plays an important role within chondrocytes by regulating multiple target genes through DNA-binding activity [15]. Furthermore, it participates in the processes of cell proliferation and differentiation [16, 17]. The role of *EGR-1* in cartilage tissue and the mechanisms affecting osteoarthritis are unclear and controversial.

Insulin-like growth factor II (IGF-II) is a growth factor with a complex structure and regulatory function [18]. IGF-II controls the expression of genes related to cartilage formation in chondrocytes, and a decrease in its expression is linked to hampered chondrocyte proliferation. Studies have demonstrated that IGF-II expression is significantly reduced in the cartilage of osteoarthritis patients [19]. In an in vitro animal inflammation model assay, increased levels of IGF-II enhanced the levels of the cartilage matrix while decreasing the expression of degradation proteins, such as matrix metallopeptidase 13 (MMP13), thereby protecting cartilage integrity [20].

The purpose of this study was to observe the effect of cyclic negative pressure within the normal physiological range on the proliferation of C28/I2 chondrocytes and to investigate the related molecular mechanisms. The results of this study would clarify the potential mechanisms behind the effect of cyclic negative pressure on cartilage health.

## Materials and methods

### Culture of chondrocytes and periodic stress intervention

The C28/I2 cell line was obtained from Qingqi Biotechnology (Shanghai, China). The cells were cultured in Dulbecco’s Modified Eagle (DMEM) High Sugar Medium (Procell, China) supplemented with 10% fetal bovine serum (FBS) (Sigma, USA) and 1% penicillin/streptomycin (Servicebio, China). Cells were cultured at 37 ^∘^C, 5% CO_2_, and 95% humidified air in an incubator for 2–3 generations. Following this, cells displaying logarithmic growth were isolated and selected for subsequent experimental research. Based on experimental requirements, the researchers designed an automatic barometric pressure control system for a biological incubator. The system is based on a Proportional–Integral–Derivative (PID) algorithm and comprises a controller, a motor, a barometric pressure sensor, and a user interface device. The air pressure sensor acquires the air pressure value inside the incubator and inside the air pressure maintenance box in real time and maintains the pressure difference between the two at a set value. The barometric pressure sensor has a measurement range of −750 to 750 mmHg (S2311GN347-1F1, Utility model patent, China) (Figure 1).

Third-generation chondrocytes were subjected to cyclic negative pressure treatment. The cells were seeded at a density of 6×10^3^ in 96-well plates and 6×10^5^ in 6-well plates before loading pressure. Based on our previous study, the appropriate negative pressure intensity was determined. The cells were then placed in a sealed cassette within a pneumatic pressure maintenance system and exposed to a pressure of −10 mmHg for 1 h. After that, the pressure was released to maintain a normobaric environment for another hour. The negative pressure group (NP Group) underwent three cyclic interventions in one day, which were repeated for a total of two days. The control group (CON Group) was a non-negative pressure group placed under the same incubation conditions.

### Detection of cell vitality and proliferation

Chondrocyte cell vitality was detected using the Cell Counting Kit-8 (CCK-8) method (MeilunBio, China). Cells were inoculated into 96-well plates at a density of 6 × 10^3^ cells per well. Chondrocytes in the NP group were subjected to two days of cyclic negative pressure intervention, while those in the CON group were cultured under normal conditions. Subsequently, CCK-8 reagent was added and incubated at 37 ^∘^C and 5% CO_2_ for 2 h. The optical density (OD) at 450 nm was detected using a microplate reader (BioTek, USA).

Cell proliferation was detected using the 5-ethynyl-2’-deoxyuridine (EdU) method and immunofluorescence. Cells were inoculated into 6-well plates according to grouping criteria for corresponding treatments, followed by continued incubation of the cells using 1× EdU working solution at 37 ^∘^C for 2 h. Cells were fixed using a 4% paraformaldehyde solution for 15 min. After washing with phosphate-buffered saline (PBS) solution containing 3% bovine serum albumin (BSA), the cells were permeabilized using PBS solution containing 0.3% Triton X-100 for 15 min. The click reaction solution was prepared according to the manufacturer’s instructions; the mixture contains the compounds required for the bonding of Alexa Fluor^®^ 555 to EdU (Epizyme Biomedical, China). The cells were incubated in the click reaction solution at room temperature, protected from light, for 30 min, followed by washing and staining of nuclei using 1× Hoechst 33342 (1:1000 in PBS) for 10 min, and washing again. Regarding the immunofluorescence imaging system, an inverted microscope (IX73, OLYMPUS, Japan) was used. EdU and nucleus images were acquired using lasers at wavelengths of 555 and 346 nm.

### RNA-sequencing for the detection of differentially expressed genes (DEGs)

RNA sequencing analysis and quantification were utilized to investigate the alterations in cellular mRNA levels after exposure to cyclic negative pressure. Three biological duplicates from each group were sent to BGI Genomics (Wuhan, China) for transcriptome sequencing. The sequencing was performed using the BGISEQ platform, with alignment to the reference genome sequence using Hierarchical Indexing for Spliced Alignment of Transcripts (HISAT). Differential gene analysis was conducted using the DESeq2 method.

The DESeqDataSetFromMatrix function was used to construct the input matrix. The data were normalized using the DESeq function, and the result function was employed to perform the differential analysis to obtain the DEGs with significant differential expression between the two groups. Specifically, if a gene’s expression level increased in the NP group compared to that in the CON group, the gene was determined to be upregulated; conversely, if the expression level of a gene decreased in the NP group compared with the CON group, the gene was considered downregulated. The screening conditions were set as |log2FoldChange| > 0.5 and *P* < 0.05.

The raw sequence data reported in this paper have been deposited in the Genome Sequence Archive (Genomics, Proteomics & Bioinformatics 2021) in the National Genomics Data Center (Nucleic Acids Research 2022), China National Center for Bioinformation/Beijing Institute of Genomics, Chinese Academy of Sciences (data number: HRA007672), and are publicly accessible at https://ngdc.cncb.ac.cn/gsa-human/.

### Analysis of the DEGs

DEGs were analyzed for Gene Ontology (GO) and Kyoto Encyclopedia of Genes and Genomes (KEGG) enrichment using the ClusterProfiler package with R version 4.3.1 software. Protein interaction network analysis of DEGs was also performed using the STRING database (https://cn.string-db.org/), and key gene screening of differential genes was conducted using the Cytoscape software (version 3.7) with the cytoHubba plugin.

### Transient transfection and RNA interference

GenePharma (Suzhou, China) designed small interfering RNA (siRNA) sequences targeting *IGF-II* and *EGR-1* were used to downregulate their expression. The siRNA sequences are shown in Table 1. The siRNA transfection was performed following the manufacturer’s instructions. The siRNA/liposome complex was added to the 6-well plates and incubated at 37 ^∘^C for at least 24 h. The knockout efficiency was confirmed by quantitative reverse transcription polymerase chain reaction (qRT-PCR) and western blotting.

**Table 1:** siRNA sequence targeting *IGF-II* and *EGR-1*

	**Sense (5’-3’)**	**Antisense (5’-3’)**
*IGF-II*-si#1	UCGAUGCUGGUGCUUCUCACCUUCU	AGAAGGUGAGAAGCACCAGCAUCGA
*IGF-II*-si#2	CGAUGCGGUGCUUCUCACCUUCUU	AAGAAGGUGAGAAGCACCAGCAUCG
*EGR-1*-si#1	GCGAUGAACGCAAGAGGCAUACCAA	UUGGUAUGCCUCUUGCGUUCAUCGC
*EGR-1*-si#2	GGACAAUUGAAAUUUGCUAAA	UAGCAAAUUUCAAUUGUCCUG
*NC*-siRNA	UUCUCCGAACGUGUCACGUTT	ACGUGACACGUUCGGAGAATT

siRNA: Small interfering RNA; *IGF-II*: Insulin-like growth factor II; *EGR-1*: Early growth response 1; NC: Negative control.

The plasmid for *EGR-1* overexpression was purchased from Fenghui Biotechnology^TM^ (China). Cells were seeded into 6-well plates and then transfected with the *h-EGR1* (NM_001964) plasmid using Lipo8000 Transfection Reagent (Beyotime Biotechnology, China). The transfection was carried out at 37 ^∘^C with 5% CO_2_ for 24–72 h. Subsequently, the overexpression of *EGR-1* was confirmed through qRT-PCR and western blot analysis.

### Gene expression analysis

Quantification of mRNA levels for collagen type II (*COL2A1*), SRY-box transcription factor 9 (*SOX9)*, *IGF-II*, and *EGR-1* was performed using qRT-PCR. Total RNA was extracted from cultured cells following the manufacturer’s instructions. The RNA from each sample was reverse transcribed into complementary DNA (cDNA) using a gDNA digester plus kit (Yeasen Biotechnology, China), and the cDNA was stored in aliquots at −20 ^∘^C until subsequent experiments. qRT-PCR was then performed using a SYBR Green mix, with the program set to initialize at 95 ^∘^C for 30 s, followed by denaturation at 95 ^∘^C for 3 s and annealing at 55 ^∘^C for 20 s, for a total of 40 cycles. The cDNA was used as a template for qRT-PCR using the CFX Connect™ Real-Time System (Bio-Rad, USA). qRT-PCR was performed using the 2^−ΔΔCT^ formula to calculate the amount of target mRNA. The specific primers used are listed in Table 2.

**Table 2:** Human gene primer sequences used for qRT-PCR

**Gene**	**Forward primers**	**Reverse primers**
*COL2A1*	TGGACGATCAGGCGAAACC	GCTGCGGATGCTCTCAATCT
*SOX9*	AGCGAACGCACATCAAGAC	CTGTAGGCGATCTGTTGGGG
*IGF-II*	CCGTGGCATCGTTGAGGAGTG	CGGGGTATCTGGGGAAGTTGTC
*EGR-1*	CCACGCCGAACACTGACATT	GAGGGGTTAGCGAAGGCTG
*GAPDH*	AATTCCATGGCACCGTCAAG	AGCATCGCCCCACTTGATTT

qRT-PCR: Quantitative reverse transcription polymerase chain reaction; *COL2A1*: Collagen type II; *SOX9*: SRY-Box transcription factor 9; *IGF-II*: Insulin-like growth factor II; *EGR-1*: Early growth response 1; *GAPDH*: Glyceraldehyde-3-phosphate dehydrogenase.

### Western blot

For both groups, the cells were rinsed with chilled PBS, then treated with protease inhibitor and phosphatase inhibitor (Beyotime Biotechnology, China) and radioimmunoprecipitation assay (RIPA) lysis buffer (Servicebio, China) for 20 min on ice. The appropriate volume of upsampling buffer was added, and the samples were denatured at elevated temperatures. A total of 15 µg of protein was added to separate the proteins using a sodium dodecyl sulfate-polyacrylamide gel electrophoresis (SDS-PAGE) gel. Subsequently, the proteins were transferred to polyvinylidene fluoride (PVDF) membranes with 3% BSA (Beyotime Biotechnology, China) closure. Primary antibodies, including *COL2A1* (1:1000) (Proteintech, China), *SOX9* (1:1000) (Proteintech, China), *IGF-II* (1:1000) (Beyotime Biotechnology, China), *EGR-1* (1:1000) (Proteintech, China), and β-actin (1:1500) (Servicebio, China), were incubated. The membranes were subsequently incubated with horseradish peroxidase (HRP)-conjugated IgG secondary antibodies (Beyotime Biotechnology, China), and protein bands were detected using an enhanced chemiluminescence (ECL) system (Bio-Rad).

### Statistical analysis

All data are from independent samples and observations. The data are presented as the mean ± standard error. One-way analysis of variance (ANOVA) followed by Dunnett’s multiple comparisons test was performed using GraphPad Prism version 9.5.0 for Windows (GraphPad Software, Boston, MA, USA, www.graphpad.com). Shapiro–Wilk tests and Student’s *t*-tests were performed using GraphPad Prism version 9.5.0 for Windows (GraphPad Software, Boston, MA, USA, www.graphpad.com) to test the normality of the data distribution and to analyze differences between the two groups. Statistical significance was defined as *P* < 0.05.

## Results

### Cyclic negative pressure treatment promotes the proliferation of chondrocytes

**Figure 2.**
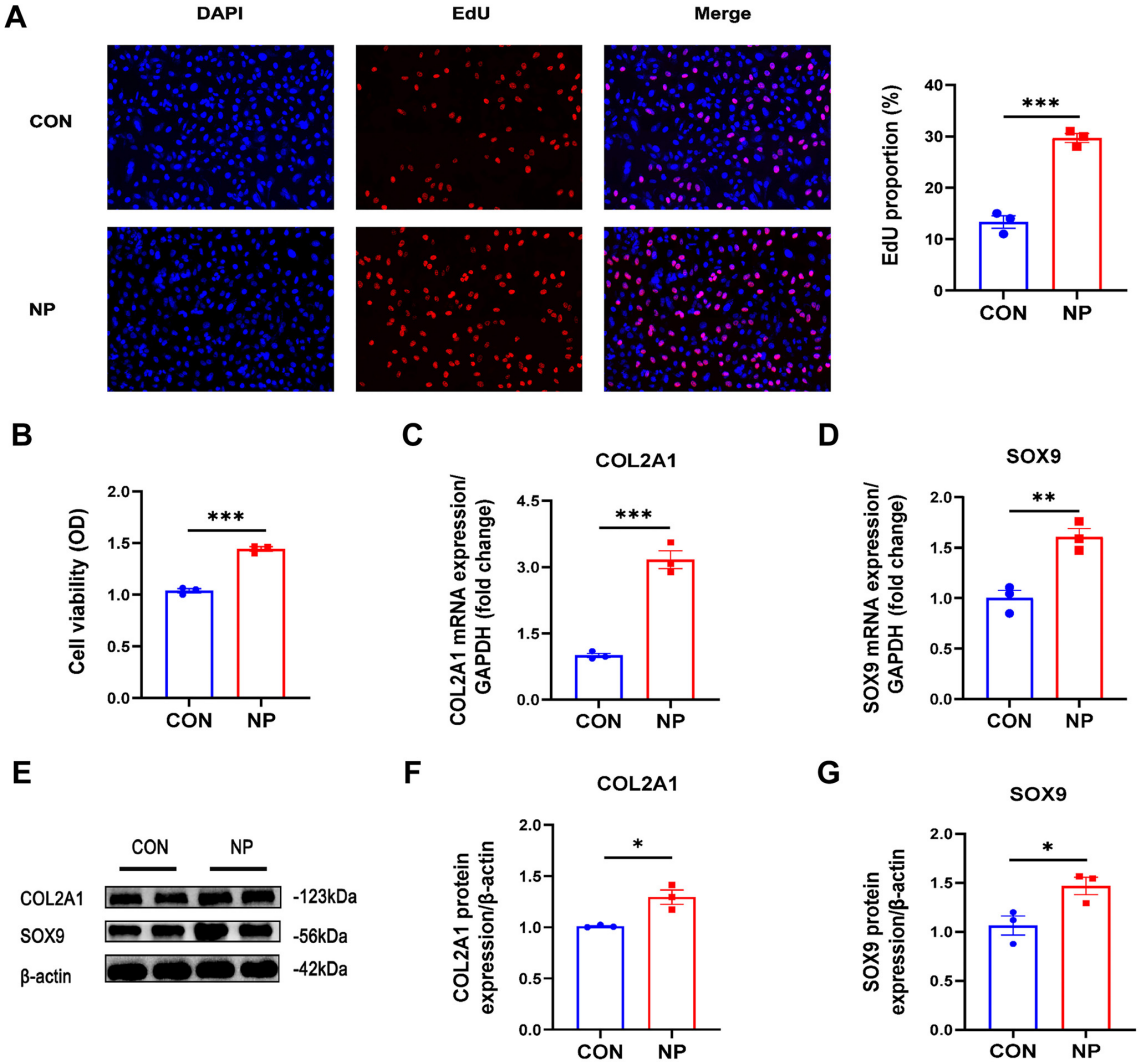
**The effects of cyclic negative pressure on chondrocyte proliferation.** (A) C28/I2 chondrocytes exhibiting proliferative changes in response to cyclic negative pressure stimulation, as demonstrated by the EdU method (*n* ═ 3); (B) CCK-8 assay displaying chondrocyte vitality (*n* ═ 3); (C) The expression of the *COL2A1* gene analyzed using qRT-PCR (*n* ═ 3); (D) The expression of the *SOX9* gene analyzed using qRT-PCR (*n* ═ 3); (E) The protein expression of COL2A1 and SOX9 analyzed through western blot; (F) A statistical histogram created to analyze the expression of the COL2A1 protein through western blot (*n* ═ 3); (G) A statistical histogram created to analyze the expression of the SOX9 protein through western blot (*n* ═ 3). *P < 0.05; **P < 0.01; ***P < 0.001; ****P < 0.0001. EdU: 5-Ethynyl-2’-deoxyuridine; CCK-8: Cell Counting Kit-8; *COL2A1*: Collagen type II; qRT-PCR: Quantitative reverse transcription polymerase chain reaction; *SOX9*: SRY-box transcription factor 9.

The results of EdU and CCK-8 experiments showed that chondrocytes subjected to cyclic negative pressure for 3 h per day for two consecutive days exhibited an increase in cell proliferation and cell vitality in the NP group compared to the normobaric CON group (*P* < 0.001) (Figure 2A and 2B). The mRNA and protein expression of the characteristic molecules COL2A1 and SOX9 in chondrocytes after cyclic negative pressure intervention were detected by qRT-PCR and protein blotting. The mRNA expression of COL2A1 and SOX9 was significantly higher in the NP group compared to the CON group (*P* ═ 0.0005, *P* ═ 0.0061, respectively) (Figure 2C and 2D). Similarly, the protein expression of COL2A1 and SOX9 was significantly elevated, and the differences were statistically significant (Figure 2E–2G).

### mRNA transcriptome sequencing reveals key mechanical stimulus stress response pathways

**Figure 3.**
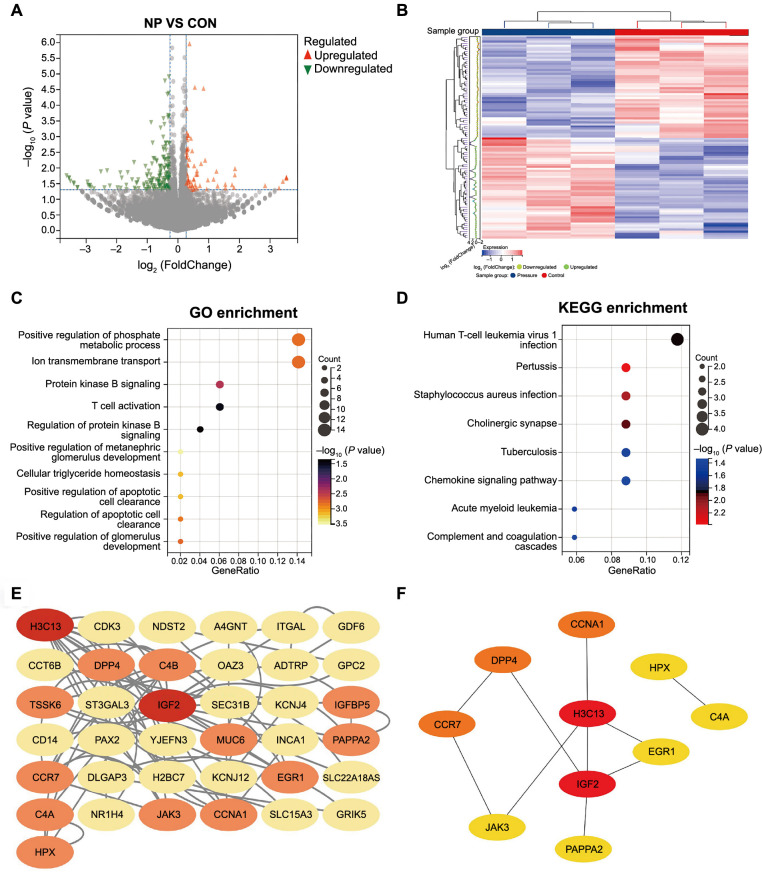
**Gene expression differences and mechanistic pathways.** (A) Volcano plot displaying the expression differences between the two groups; |log2FoldChange| > 0.5; *P* < 0.05; (B) A hierarchical clustering heatmap showcasing the related genes between the CON and NP groups. Each group included three samples: Pressure-1, Pressure-2, and Pressure-3 for the NP group, and CON-1, CON-2, and CON-3 for the CON group. The CON group did not receive cyclic negative pressure treatment, while the NP group did (*n* ═ 3); (C) GO annotation revealing the most enriched terms; *P* < 0.05; (D) KEGG annotating the forward results; *P* < 0.05; (E) The STRING database searches for interactions among DEGs; (F) The top ten differentially expressed genes retrieved using Cytoscape. CON: Control; NP: Negative pressure; GO: Gene Ontology; KEGG: Kyoto Encyclopedia of Genes and Genomes; STRING: Search Tool for the Retrieval of Interacting Genes/Proteins; DEGs: Differentially expressed genes.

RNA sequencing data revealed differential gene expression in chondrocytes from the CON and NP groups. A total of 209 DEGs were identified, of which 64 genes were upregulated and 145 genes were downregulated (Figure 3A and 3B). We performed the GO functional annotation analysis of the DEGs, including molecular functions (Figure S1A), biological processes (Figure S1B), and cellular components (Figure S1C). The top-ranked GO functional annotations were mainly related to cellular receptor signaling pathways and phosphorylated protein modification, suggesting that cell viability and proliferation were promoted in the NP group (Figure 3C). The KEGG-enriched pathway analysis showed associations with the chemokine signaling pathway and oxidative phosphorylation, indicating potential mechanistic pathways affected by cyclic negative pressure (Figure 3D). Protein–protein interaction (PPI) network analysis was performed on the DEGs, and the top ten key genes were screened. Among these genes, *IGF-II*, which is positively associated with chondrocyte proliferation, was selected (Figure 3E and 3F).

**Figure 4.**
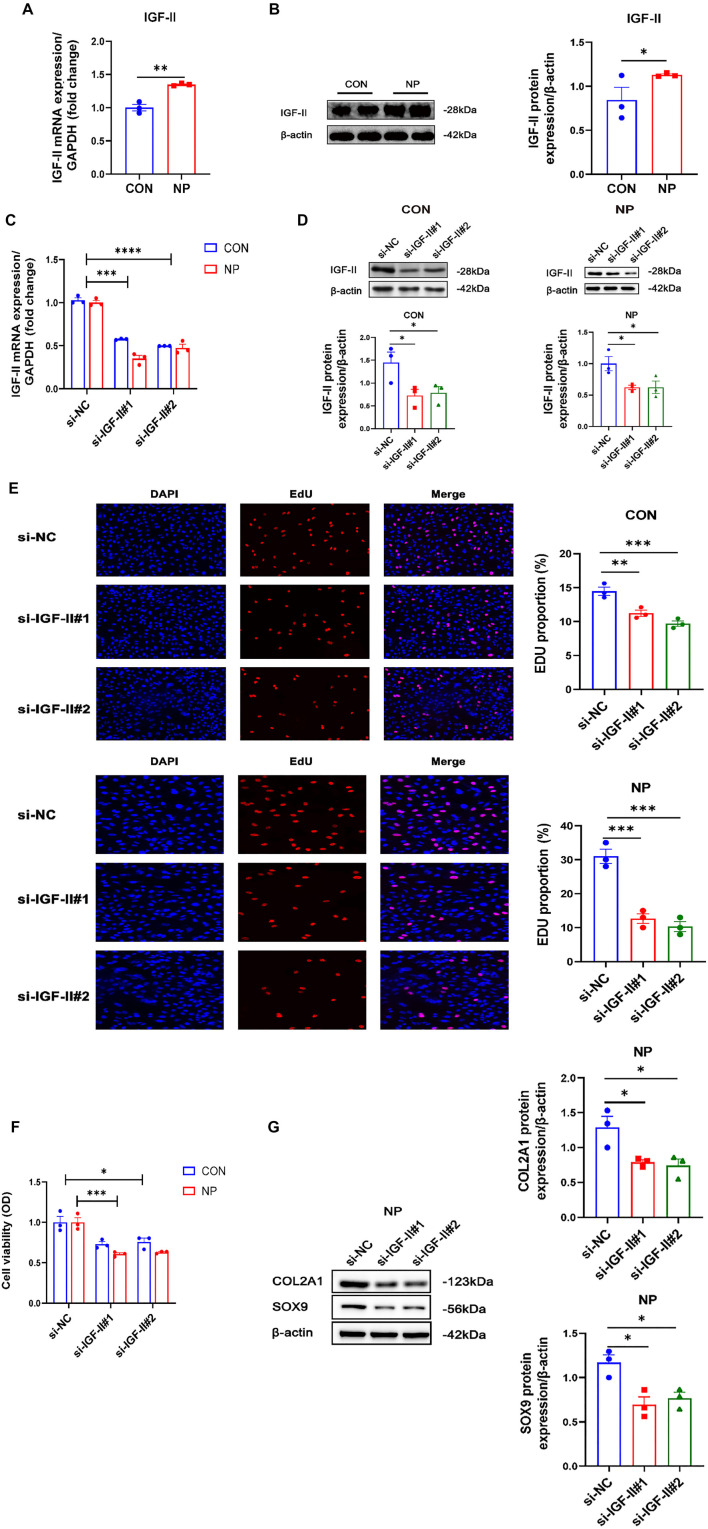
***IGF-II* molecules as key molecules in cyclic negative pressure affecting cell proliferation.** (A) Showcasing the qRT-PCR analysis of IGF-II (*n* ═ 3); (B) Displaying the western blot analysis of IGF-II molecule expression and statistical histograms (*n* ═ 3); (C) qRT-PCR analysis of the expression of IGF-II mRNA in CON and NP groups after transfection with negative control and si-IGF-II, respectively (*n* ═ 3); (D) Western blot analysis of IGF-II molecule expression in cells after transfection of si-NC and si-IGF-II in CON and NP groups (*n* ═ 3); (E) EdU method used to detect cell proliferation ability after transfection of si-NC and si-IGF-II in CON and NP groups (*n* ═ 3); (F) CCK-8 assay used to detect the vitality of cells treated with si-NC and si-IGF-II (*n* ═ 3); (G) Western blot analysis of the extracellular matrix molecule expression in chondrocytes from the NP group after the knockdown of *IGF-II* (*n* ═ 3). *P < 0.05; **P < 0.01; ***P < 0.001; ****P < 0.0001. qRT-PCR: Quantitative reverse transcription polymerase chain reaction; IGF-II: Insulin-like growth factor II; CON: Control; NP: Negative pressure; EdU: 5-Ethynyl-2’-deoxyuridine; CCK-8: Cell Counting Kit-8.

### Cyclic negative pressure significantly promotes IGF-II gene expression

Elevated expression of the *IGF-II* gene was confirmed in the NP group compared to the CON group by both qRT-PCR (*P* < 0.01) and western blot assay (*P* < 0.05) (Figure 4A and 4B). To investigate the effect of IGF-II on chondrocytes under cyclic negative pressure intervention, we transfected the cells with si-RNA and added si-IGF-II mixed with liposomes to the cell culture medium. The results showed that, in the NP group, compared with the si-NC-treated chondrocytes, the IGF-II mRNA expression of si-IGF-II-treated chondrocytes was significantly decreased (*P* < 0.0001) (Figure 4C).

Additionally, the protein expression of IGF-II in chondrocytes from the CON and NP groups was examined, and the results showed a significant decrease in IGF-II protein expression in chondrocytes of both groups after si-IGF-II treatment (*P* < 0.05) (Figure 4D). EdU results indicated that cell proliferation ability was significantly reduced in si-IGF-II-treated CON and NP groups (*P* < 0.01; *P* < 0.001; respectively) (Figure 4E). Moreover, the cellular vitality of chondrocytes in the CON and NP groups after siRNA treatment was assessed, revealing that knockdown of *IGF-II* significantly reduced the cell vitality of chondrocytes (*P* < 0.05; *P* < 0.001; respectively) (Figure 4F).

Furthermore, the expression of extracellular matrix proteins in chondrocytes of the NP group was examined at the protein level, showing that the expression of COL2A1 and SOX9 proteins were significantly decreased in chondrocytes treated with si-IGF-II (*P* < 0.05) (Figure 4G). Thus, silencing of *IGF-II* negated the effect of cyclic negative pressure on cell proliferation, suggesting that cyclic negative pressure further promoted chondrocyte proliferation and extracellular matrix synthesis by increasing the expression level of *IGF-II*.

### EGR-1 transcriptional regulation of IGF-II activity leads to cyclic negative pressure promoting chondrocyte proliferation

**Figure 5.**
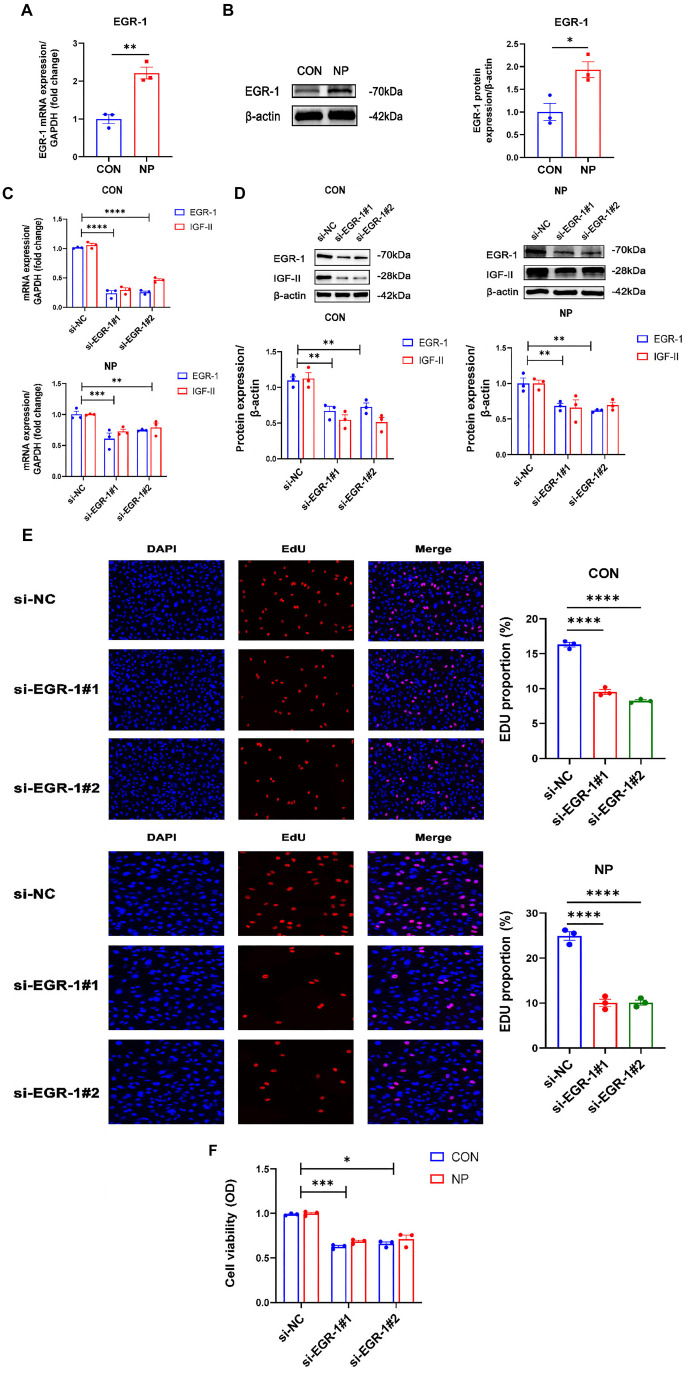
**Validation of *EGR-1* and effects on *IGF-II* after knockdown of *EGR-1*.** (A) qRT-PCR analysis of EGR-1 (*n* ═ 3); (B) Western blot analysis of EGR-1 molecule expression (*n* ═ 3); (C) mRNA expression of EGR-1 and IGF-II in chondrocytes transfected with si-NC and si-EGR-1, respectively, in CON and NP groups (*n* ═ 3); (D) Western blot analysis of EGR-1 and IGF-II protein expression in chondrocytes after transfection of si-NC and si-EGR-1 in CON and NP groups, respectively (*n* ═ 3); (E) EdU method used to detect the proliferative capacity of cells in CON and NP groups after treatment with si-NC and si-EGR-1 (*n* ═ 3); (F) CCK-8 assay used to detect the vitality of cells treated with si-NC and si-EGR-1 (*n* ═ 3). *P < 0.05; **P < 0.01; ***P < 0.001; ****P < 0.0001. qRT-PCR: Quantitative reverse transcription polymerase chain reaction; EGR-1: Early growth response 1; IGF-II: Insulin-like growth factor II; NC: Negative control; CON: Control; NP: Negative pressure; EdU: 5-ethynyl-2’-deoxyuridine; CCK-8: Cell Counting Kit-8.

To investigate the molecular mechanism of *IGF-II* activation by cyclic negative pressure, we selected *EGR-1*, which is linked to *IGF-II*, through PPI network mapping and related literature queries. Our transcriptome sequencing data indicated that *EGR-1* was upregulated in the NP group. We validated this finding at both mRNA and protein levels, showing that cyclic negative pressure promotes *EGR-1* expression (*P* < 0.01; *P* < 0.05; respectively) (Figure 5A and 5B). Our next study will be conducted on *EGR-1* and explore its link with *IGF-II* in cartilage.

To elucidate the effect of *EGR-1* on the increase in *IGF-II* induced by cyclic negative pressure, cells treated with EGR-1 siRNA were subjected to cyclic negative pressure intervention. The data indicated that both groups of EGR-1 siRNA-treated cells exhibited a significant reduction in mRNA levels compared to negative control (si-NC) cells. Similarly, there was a significant decrease in mRNA expression of IGF-II (Figure 5C). At the protein level, the knockdown of EGR-1 significantly decreased the expression of EGR-1 and IGF-II in chondrocytes of the CON and NP groups (*P* < 0.01) (Figure 5D). Cell proliferation assays of cells transfected with siRNA in the CON and NP groups were performed using the EdU assay. The results showed that cells treated with si-EGR-1 exhibited a significant decrease in their cell proliferation ability (*P* < 0.0001) (Figure 5E). Furthermore, CCK-8 data showed that the cell vitality of chondrocytes was significantly reduced when cyclic negative pressure was applied and *EGR-1* was knocked down (*P* < 0.001) (Figure 5F).

**Figure 6.**
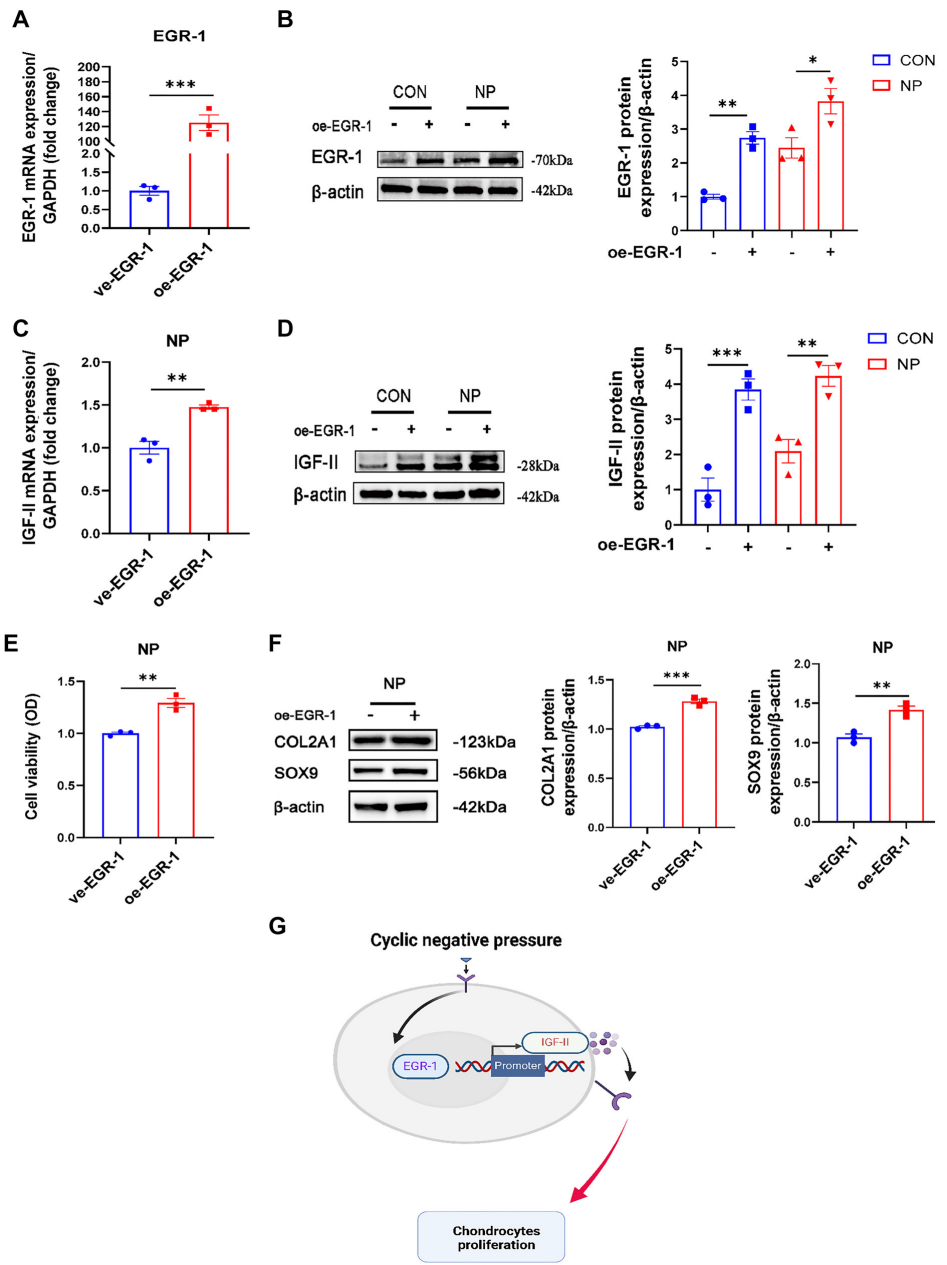
**Effect of overexpression of *EGR-1* on *IGF-II* and cell proliferation.** (A) qRT-PCR analysis of overexpression of *EGR-1* (*n* ═ 3); (B) Western blot analysis after overexpression of EGR-1 in CON and NP groups (*n* ═ 3); (C) mRNA expression of IGF-II after overexpression of EGR-1 in cells of CON and NP groups (*n* ═ 3); (D) IGF-II protein expression after overexpression of *EGR-1* in cells of CON and NP groups (*n* ═ 3); (E) CCK-8 assay used to detect the vitality of cells treated with overexpressed *EGR-1* (*n* ═ 3); (F) Western blot analysis of extracellular matrix components of chondrocytes in the NP group after overexpression of *EGR-1* (*n* ═ 3); (G) Schematic representation of how cyclic negative pressure promotes chondrocyte proliferation by affecting *EGR-1* and *IGF-II* together (https://biorender.com/). *P < 0.05; **P < 0.01; ***P < 0.001. qRT-PCR: Quantitative reverse transcription polymerase chain reaction; EGR-1: Early growth response 1; CON: Control; NP: Negative pressure; IGF-II: Insulin-like growth factor II; CCK-8: Cell Counting Kit-8.

In contrast, cells that overexpress *EGR-1* (oe-*EGR-1)* showed a significant increase in mRNA levels, approximately 100-fold higher than cells transfected with the empty plasmid (ve-*EGR-1*) (*P* < 0.001) (Figure 6A), and this increase was also significant at the protein level (Figure 6B). In the NP group, overexpression of *EGR-1* led to a significant upregulation of both mRNA and protein expression of IGF-II (Figure 6C and 6D). Regarding cell vitality, CCK-8 data showed that cells overexpressing *EGR-1* followed by cyclic negative pressure intervention exhibited a significant increase in vitality (*P* < 0.01) (Figure 6E). Moreover, the expression of extracellular matrix proteins in chondrocytes from the NP group was examined at the protein level, revealing that the expression of both COL2A1 and SOX9 proteins in chondrocytes was significantly upregulated after overexpression of *EGR-1* (Figure 6F). Thus, we confirmed that cyclic negative pressure is involved in the cell proliferation process by inducing the expression of the *EGR-1* gene, which in turn promotes the expression of the target gene *IGF-II*, which is positively correlated with *EGR-1* (Figure 6G).

## Discussion

A proper biomechanical environment is essential for maintaining the structure and function of cartilage tissue. It has now been shown that cyclic stress increases the production of cartilage matrix, while excessive static stress significantly reduces collagen synthesis in cartilage [21–23]. In our previous clinical study, we observed a significant difference in the negative pressure values within the knee joint cavity between healthy volunteers and those with KOA. We found that the pressure within the knee joint cavity exhibited cyclic changes [14]. In this study, we cultured cells at an ambient pressure (approximately equal to 1 atmosphere) as a CON Group. We then used in vitro assays to confirm that chondrocytes cultured under conditions that simulated the negative pressure found in healthy human joints exhibited a higher proliferative effect. Additionally, the expression of *COL2A1* and *SOX9*, genes associated with cartilage health and the regulation of cartilage growth, was increased. These findings suggest that cyclic negative pressure may be beneficial for cartilage health, indicating that maintaining a certain level of negative pressure in human joints could be advantageous. The chondrocytes of the two groups were further analyzed by transcriptome sequencing. Bioinformatics methods were applied to analyze the data to obtain the differential genes between the two groups, and the *IGF-II* and *EGR-1* genes, which were highly expressed in the NP group, were identified as the focus of the study. Numerous studies have shown that *IGF-II* plays a key role in maintaining cartilage and joint homeostasis as well as promoting bone growth. *IGF-II* activates phosphoinositide 3-kinase (PI3K)/protein kinase B (Akt) and transforming growth factor-beta (TGF-β) signaling pathways in the growth plate to promote endochondral osteogenesis. In skeletal muscle, an amplification cascade occurs with autocrine IGF-II synergizing with myoblast determination protein (MyoD) to promote muscle differentiation [24–27].

Normal chondrocytes secrete IGF-II, while in pathological states, the expression level of *IGF-II* is reduced, leading to cartilage matrix damage and subsequent chondrocyte loss [19]. We found that the expression of *IGF-II* in chondrocytes in the cyclic NP group was significantly higher compared with cells cultured in normobaric pressure by RNA-seq analysis, which is consistent with the results of the above studies, suggesting that cyclic negative pressure favored chondrocyte growth. Our inhibition of *IGF-II* reduced cyclic negative pressure-induced chondrocyte proliferation and extracellular matrix synthesis, suggesting that *IGF-II* plays an important role in the promotion of chondrocyte proliferation by cyclic negative pressure.

Our next focus is to investigate how cyclic negative pressure induces elevated *IGF-II* expression. RNA-seq analysis showed that the expression of *EGR-1* was significantly elevated in chondrocytes in the cyclic NP group, which drew our attention. The activation of early genes is the first response when the external environment is altered. EGR-1 is a stress-responsive protein whose expression is up-regulated when the cell is exposed to drugs, inflammation, or growth factors [28, 29]. Studies on the correlation between *IGF-II* and *EGR-1* have not been carried out in the field of cartilage. However, in the field of oncology, high expression of *EGR-1* was found to drive the expression of growth factors, including *IGF-II*. Furthermore, *IGF-II* has been found to act as a physiological target of *EGR-1*, and the promoter region of *IGF-II* contains multiple sites that can bind to *EGR-1* or its ligands, suggesting that the nature of the *IGF-II-EGR-1* linkage is the regulation of an autocrine process [30–32]. However, existing studies provide conflicting accounts regarding the expression and specific role of *EGR-1* in osteoarthritis. On the one hand, some results show that *EGR-1* is abnormally highly expressed in osteoarthritic cartilage, and its ectopic expression exacerbates the degradation of the cartilage matrix in vivo. On the other hand, other studies report that the expression of the *EGR-1* gene in normal articular cartilage is higher than that in osteoarthritic cartilage and that it may be involved in the process of chondrogenesis [16, 33, 34]. *IGF-II*, on the contrary, has been shown by many studies to promote chondrocyte synthesis and to promote osteoblast proliferation and differentiation, ameliorating matrix damage in osteoarthritis [20, 35, 36]. In our study, we found that inhibiting or overexpressing *EGR-1* had distinct effects on the expression of *IGF-II* and the proliferation of chondrocytes under cyclic negative pressure stimulation. When *EGR-1* expression was down-regulated under cyclic negative pressure stimulation, the level of *IGF-II* expression decreased, along with a reduction in chondrocyte proliferation. Conversely, the overexpression of *EGR-1* led to increased *IGF-II* expression and further enhanced chondrocyte proliferation. These findings indicate that *IGF-II* expression is positively correlated with the level of *EGR-1* during the stimulation of chondrocytes by cyclic negative pressure.

Although there are important discoveries revealed by these studies, there are also limitations. To ensure the stability and reproducibility of our in vitro experiments, we selected the C28/I2 cell line. However, now that we have explored a well-established experimental system, an in vivo experimental model can be set up to delve deeper into the underlying mechanisms and supplement this study. Moreover, by following the methodology presented in this study on healthy human chondrocytes, the biological changes of diseased chondrocytes or tissue progenitor cells can also be explored.

In summary, our study demonstrates that cyclic negative pressure within the normal joint pressure range has a pro-proliferative effect on C28/I2 chondrocytes. For the first time, we propose a positive correlation between *IGF-II* and *EGR-1* in chondrocytes, suggesting that the combination of *IGF-II* and *EGR-1* is an important link in the response of chondrocytes to changes in the negative pressure environment.

## Conclusion

In this study, we cultured and observed the proliferation of human C28/I2 chondrocytes in vitro using a device that simulated the negative pressure conditions found in healthy human joints. We found that chondrocytes cultured under cyclic negative pressure demonstrated enhanced proliferation and cell vitality. These results suggest that cyclic negative pressure may be beneficial for maintaining cartilage health. Moreover, the positive correlation between cyclic negative pressure and the high expression of *EGR-1* and *IGF-II* suggests that *EGR-1* may be involved in the regulation of *IGF-II* activation in cartilage. This is the first time that a positive correlation between *EGR-1* and *IGF-II* expression has been identified in chondrocytes. The results of this study provide insights into the pathogenesis of articular cartilage degeneration, KOA prevention and control, and cartilage tissue engineering.

## Supplemental data

**Figure S1.**
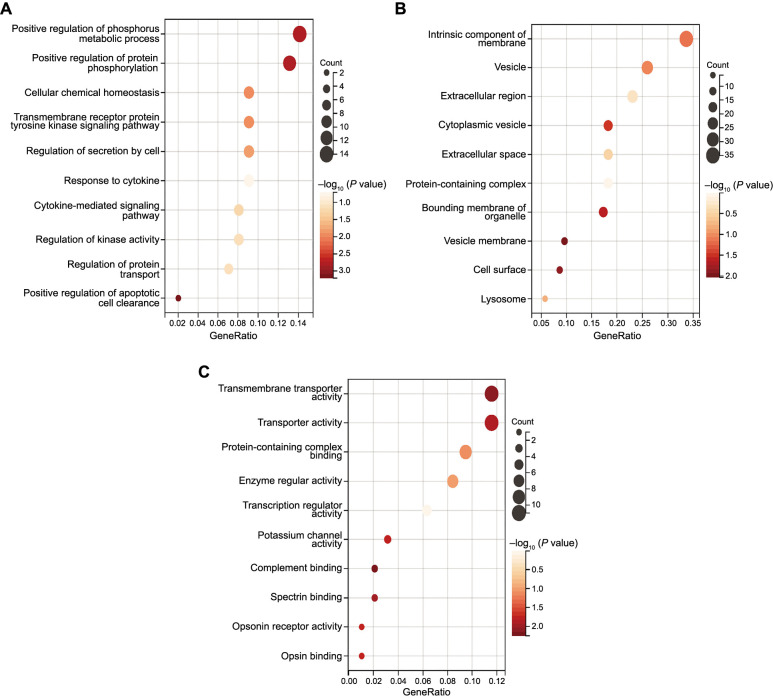
**GO functional annotation analysis of the differentially expressed genes.** (A) GO functional annotation analysis of the molecular function; (B) GO functional annotation analysis of the biological processes; (C) GO functional annotation analysis of the cellular components. GO: Gene Ontology.

## Data Availability

Data are contained within the article and supplementary materials. RNA-seq data will be shared by the corresponding authors upon reasonable request.
